# Maintenance of Sertoli Cell Number and Function in Immature Human Testicular Tissues Exposed to Platinum-Based Chemotherapy—Implications for Fertility Restoration

**DOI:** 10.3389/ftox.2022.825734

**Published:** 2022-03-21

**Authors:** Gabriele Matilionyte, Melissa D. Tharmalingam, Iris Sanou, Federica Lopes, Sheila Lane, Jan-Bernd Stukenborg, Norah Spears, Richard A. Anderson, Rod T. Mitchell

**Affiliations:** ^1^ MRC Centre for Reproductive Health, Queen’s Medical Research Institute, The University of Edinburgh, Edinburgh, United Kingdom; ^2^ KK Women’s and Children’s Hospital, Edinburgh, Singapore; ^3^ Medical School, University of Dundee, Dundee, United Kingdom; ^4^ Department of Womens and Reproductive Health, University of Oxford and Oxford University Hospitals NHS Foundation Trust, Edinburgh, United Kingdom; ^5^ NORDFERTIL Research Lab Stockholm, Childhood Cancer Research Unit, Department of Women’s and Children’s Health, Karolinska Institutet, Karolinska University Hospital, Solna, Sweden; ^6^ Biomedical Sciences, The University of Edinburgh, Edinburgh, United Kingdom; ^7^ Royal Hospital for Children and Young People, Edinburgh, United Kingdom

**Keywords:** human, testis, cisplatin, carboplatin, sertoli cells, pre-pubertal, fetal, fertility

## Abstract

**Background:** Retrospective studies in adult survivors of childhood cancer show long-term impacts of exposure to alkylating chemotherapy on future fertility. We recently demonstrated germ cell loss in immature human testicular tissues following exposure to platinum-based chemotherapeutic drugs. This study investigated the effects of platinum-based chemotherapy exposure on the somatic Sertoli cell population in human fetal and pre-pubertal testicular tissues.

**Methods:** Human fetal (*n* = 23; 14–22 gestational weeks) testicular tissue pieces were exposed to cisplatin (0.5 or 1.0 μg/ml) or vehicle for 24 h *in vitro* and analysed 24–240 h post-exposure or 12 weeks after xenografting. Human pre-pubertal (*n* = 10; 1–12 years) testicular tissue pieces were exposed to cisplatin (0.5 μg/ml), carboplatin (5 μg/ml) or vehicle for 24 h *in vitro* and analysed 24–240 h post-exposure; exposure to carboplatin at 10-times the concentration of cisplatin reflects the relative clinical doses given to patients. Immunohistochemistry was performed for SOX9 and anti-Müllerian hormone (AMH) expression and quantification was carried out to assess effects on Sertoli cell number and function respectively. AMH and inhibin B was measured in culture medium collected post-exposure to assess effects on Sertoli cell function.

**Results:** Sertoli cell (SOX9^+ve^) number was maintained in cisplatin-exposed human fetal testicular tissues (7,647 ± 459 vs. 7,767 ± 498 cells/mm^2^; *p* > 0.05) at 240 h post-exposure. No effect on inhibin B (indicator of Sertoli cell function) production was observed at 96 h after cisplatin (0.5 and 1.0 μg/ml) exposure compared to control (21 ± 5 (0.5 μg/ml cisplatin) vs. 23 ± 7 (1.0 μg/ml cisplatin) vs. 25 ± 7 (control) ng/ml, *p* > 0.05). Xenografting of cisplatin-exposed (0.5 μg/ml) human fetal testicular tissues had no long-term effect on Sertoli cell number or function (percentage seminiferous area stained for SOX9 and AMH, respectively), compared with non-exposed tissues. Sertoli cell number was maintained in human pre-pubertal testicular tissues following exposure to either 0.5 μg/ml cisplatin (6,723 ± 1,647 cells/mm^2^) or 5 μg/ml carboplatin (7,502 ± 627 cells/mm^2^) compared to control (6,592 ± 1,545 cells/mm^2^).

**Conclusions:** This study demonstrates maintenance of Sertoli cell number and function in immature human testicular tissues exposed to platinum-based chemotherapeutic agents. The maintenance of a functional Sertoli cell environment following chemotherapy exposure suggests that fertility restoration by spermatogonial stem cell (SSC) transplant may be possible in boys facing platinum-based cancer treatment.

## 1 Introduction

Development of improved cancer therapies have resulted in long-term survival (more than 5 years) in over 80% of childhood cancer patients (Anderson et al. (2015)). Although life-saving, it is well recognised that chemotherapeutic treatment during childhood has implications on survivors’ health in adulthood; one of the long-lasting side effects of cancer treatment is infertility (Anderson and Wallace (2016)). Anti-metabolites, vinca alkaloids and topoisomerase inhibitors fall within a low or moderate gonadotoxicity risk group, whilst alkylating agents and platinum-based “alkylating-like” agents are considered to be highly gonadotoxic (Allen et al. (2018)). Dose-dependent gonadotoxicity can be estimated using a Cyclophosphamide Equivalent Dose (CED) and there is some correlation between CED and risk of azoospermia in adult survivors of childhood cancer. However, platinum-based alkylating-like agents are not included in the calculation (Green et al., 2014a; Green et al., 2014b).

Cisplatin and carboplatin are platinum-based alkylating-like chemotherapeutic drugs that are commonly used in paediatric oncology (Dasari and Tchounwou (2014)). Cisplatin (first generation) is widely used to treat childhood carcinomas, germ cell tumors, lymphomas and sarcomas (Dasari and Tchounwou (2014)). Carboplatin (second generation) has been developed with the aim of overcoming serious cisplatin-induced side effects such as ototoxicity and nephrotoxicity (Dasari and Tchounwou (2014)). Cisplatin and carboplatin share a similar structure (central platinum ion surrounded by four ligands), however they have different pharmacokinetics requiring 4–10 times higher dose of carboplatin to achieve the same cytotoxic result to the cancer cells (Malhotra and Perry (2003); Goodsell (2006); Dasari and Tchounwou (2014)).

The retrospective follow-up of what is currently the largest childhood cancer survivor cohort shows that both men and women who received cisplatin as part of childhood cancer treatment have a lower chance of pregnancy when they are adults compared to their healthy same-sex siblings (Chow et al., 2016). Chemotherapy-induced gonadotoxicity in males could be a result of either direct damage to the germ cells or indirect damage to somatic cell populations (most likely the Sertoli cell population), which are consequently unable to support the germ cells (Anderson et al. (2015)). Recently we have shown that experimental exposure to cisplatin or carboplatin leads to acute reduction in germ cell (gonocyte and (pre)spermatogonial) numbers in immature human testicular tissues (Tharmalingam et al., 2020). However, it remains to be determined whether exposure to platinum-based alkylating-like chemotherapeutic agents also affects the somatic cell environment.

Sertoli cell number, function and maturity are crucial in providing the support and regulating the differentiation of germ cells. Sertoli cells play key roles throughout testicular development, from fetal to adult stages. During fetal life, Sertoli cells are the first cell type to commit to male fate and consequently they regulate the differentiation of other testicular cell types in the developing gonad. During early post-natal life, the testicular volume increases up to 6-times due to high proliferation of Sertoli cells; it is estimated that Sertoli cells account for approximately 95% of the cellular content present in seminiferous tubules of the infant testis (Chemes (2001)). During peri-puberty, Sertoli cells undergo a second wave of proliferation and this is crucial in establishing the final Sertoli cell number that will be present in the adult testis; the final number of Sertoli cells directly determines the spermatogenic capability of the adult testis (Sharpe et al. (2003)).

Sertoli cell function can be determined by measuring two key hormones, anti-Müllerian hormone (AMH) and inhibin B. Levels of AMH increase during fetal life and AMH is responsible for inducing the regression of Müllerian ducts that would give rise to female reproductive organs (Aksglaede et al., 2010). Levels of AMH fall to low levels (approximately 3–4% of the concentration secreted during fetal life) during puberty and adulthood as the Sertoli cells mature. Therefore, AMH is often used as a “read-out” of Sertoli cell maturity (Aksglaede et al., 2010; Hutka et al., 2020). Inhibin B is another key hormone secreted by Sertoli cells, proposed as an indirect indicator of normal testicular growth, Sertoli cell number and a possible predictor of future reproductive capability of the immature testis (Andersson and Skakkebaek, 2001; Bergada et al., 2006). In addition, a correlation between levels of inhibin B and sperm concentration has been shown in samples taken from men with normal and abnormal sperm counts, therefore, acting as an indicator of spermatogenesis in pubertal and adult testis (Anderson et al., 1997; Hipler et al., 2001).

Sertoli cells, located within seminiferous cords/tubules in close proximity with germ cells, are particularly important to consider when assessing gonadotoxicity of chemotherapeutic drugs, especially when considering fertility preservation options in childhood cancer patients receiving gonadotoxic treatment. For these patients, it could theoretically be possible to restore fertility by SSC transplantation from cryopreserved testis tissue (Anderson et al. (2015)). However, this is dependent on maintained Sertoli cell number and function to support spermatogenesis in the transplanted testis. Having previously shown that exposure to platinum-based chemotherapeutic agents led to reduction in germ cell number, we aimed to determine how exposure to clinically-relevant concentrations of cisplatin or carboplatin affects Sertoli cell number, function and maturation in human fetal and pre-pubertal testicular tissues.

## 2 Materials and Methods

### 2.1 Experimental Design

Human fetal testicular samples were randomly selected from previous experiments in which exposure to cisplatin or carboplatin was shown to induce acute germ cell reduction (Tharmalingam et al., 2020). This included human fetal testicular tissues cultured for 24, 72, 96 and 240 h post-exposure, some of which that were further xenografted for 12 weeks and human pre-pubertal testicular tissues that were cultured for 24 and 96 h post-exposure were included in this study. Additional experiments using samples of human pre-pubertal testicular tissues for 72 and 240 h post-exposure were set up for this study. Experimental design is extensively described in Tharmalingam et al. (2020).

### 2.2 Study Approval

Use of human fetal testicular tissues for research was approved by the South East Scotland Research Ethics Committee (LREC08/S1101/1), NRES committee North East–Newcastle and North Tyneside 1 (08/H0906/21 + 5) and NRES Committee London–Fulham (18/10/0822). Written informed consent was obtained from the pregnant women. Collection and use of human pre-pubertal testicular tissues in research was approved by the South East Scotland Research Ethics Committee (13/SS0145) and Oxford University Hospitals NHS Foundation Trust (2016/0140). Patients’ parents/guardians and/or the patient themselves (where appropriate) gave written informed consent prior to undergoing a testicular biopsy for fertility preservation.

For experiments involving animals, specific experimental and ethical approval was obtained from the United Kingdom Home Office. All procedures were performed by the University of Edinburgh Bioresearch and Veterinary Services and carried out in accordance with the Animal (Scientific procedures) Act 1986.

### 2.3 Tissue Collection

#### 2.3.1 Human Fetal Testicular Tissues

Second trimester human fetuses (14–22 weeks gestation; total *n* = 23) were obtained from medical and surgical elective terminations of pregnancy. None of the terminations were due to fetal abnormalities. Gestational age was determined by ultrasound scan and subsequent direct measurement of foot length. Gonads were collected and PCR for the male-specific SRY gene was performed to determine the fetal sex.

#### 2.3.2 Pre-Pubertal Human Testicular Tissues

Pre-pubertal testicular tissue biopsies (*n* = 10 patients; aged 1–12 years, details in Table 1) were obtained from patients undergoing a testicular biopsy for fertility preservation prior to receiving chemotherapy treatment at the Royal Hospital for Sick Children in Edinburgh and Oxford University Hospitals NHS foundation Trust in Oxford. For research purposes, a small portion (∼10%) of the sample was taken. Once collected, the testicular tissue from Edinburgh was placed in Nutristem^®^ hPSC XF Medium (Biological industries) supplemented with 1% penicillin/streptomycin (Sigma-Aldrich) for immediate transportation to the research facilities. Testicular biopsies from Oxford were placed in Hank’s Balanced Salt solution (VWR) with 10% human serum albumin (Bio Products Laboratory) for overnight transportation at 4°C to Edinburgh research facilities.

**TABLE 1:** Pre-pubertal patient ages and diagnoses.

Patient age	Diagnosis
1 year	Ependymoma
1 year	Ependymoma
2 years	*β*-thalassemia
4 years	Neuroblastoma
4 years	*β*-thalassemia
7 years	Aplastic anaemia
8 years	Ewing’s sarcoma
8 years	Pilocytic astrocytoma
11 years	Congenital neutropenia
12 years	Pleomorphic astrocytoma

### 2.4 Hanging Drop Culture System

A hanging drop culture system (Jorgensen et al., 2014) was utilised for *in vitro* culture and drug exposure of human fetal and pre-pubertal testicular tissues. Testicular tissue samples were cut into small pieces (∼1 mm^3^) and placed into 30 μl droplets of appropriate media (for fetal tissue: Alpha MEM (Lonza); 10% fetal bovine serum, 1% penicillin/streptomycin, 1% non-essential amino acids, 2 mM L-glutamine, 2 mM sodium pyruvate and 1% insulin transferrin selenium (ITS) (all Sigma-Aldrich); for pre-pubertal tissue: Alpha MEM (Lonza) and 10% Knockout Serum Replacement (KSR; Gibco)) on the lid of a Petri dish, which was then inverted. A small volume (10 ml) of Phosphate Buffered Saline (Corning) was poured into the bottom of the dish to maintain the humidity. Dishes were incubated at 37°C (fetal tissue) and 35°C (pre-pubertal tissue) under 5% CO_2_.

Tissue pieces were cultured in regular culture medium for 1–3 days (depending on the tissue arrival) prior to exposure to treatment medium containing cisplatin (0.5 or 1.0 μg/ml; Sigma-Aldrich); carboplatin (5.0 μg/ml; Calbiochem)) or vehicle (ddH_2_0) for 24 h. Following exposure, tissue pieces were transferred and cultured in fresh drug-free culture medium until the experiment was ended at 24, 96, 72 or 240 h post-exposure.

### 2.5 Media Collection and Hormonal Assays

Drug-free tissue culture medium was collected at 24 and 96 h post-cisplatin exposure. Medium from technical replicates from each individual fetus was pooled for each treatment separately.

ELISA assays were used to assess inhibin B and AMH in culture medium (diluted 1:50) as described below.

Inhibin B gen II ELISA three-step sandwich assay kit (Beckman Coulter: A81301) was used to detect the concentration of inhibin B. Experimental samples were incubated on a shaking plate (600–800 rpm) at RT with inhibin B Gen II antibody-biotin conjugate solution for 1 h. After washes, Inhibin B gen II streptavidin-enzyme conjugate was added for 30 min, followed by TMB chromogen solution incubation for 8 min. Plates were analyzed using an Absorbance Microplate Reader (LT4500, Labtech), with filter 450 nm.

An electrochemiluminescence immunoassay was used to determine the concentration of AMH using Elecsys AMH Plus kit (Cobus: Roche, 07957190190). Incubation with a biotinylated monoclonal AMH-specific antibody, and a monoclonal AMH-specific antibody was used to form a sandwich complex, followed by a second incubation with streptavidin-coated microparticles. The reaction mixture was added into the measuring cell where the microparticles were magnetically bound on the surface of the electrode. Application of a voltage to the electrode then induced chemiluminescent emission which was measured by a photomultiplier. Results were determined via a calibration curve which the instrument specifically generated by 2-point calibration and a master curve provided via the reagent barcode.

### 2.6 Human Tissue Xenografting

Testicular tissue pieces from human fetuses (*n* = 4) were cultured and exposed to cisplatin (0.5 μg/ml) *in vitro* as described above and subsequently used for xenografting at 24 h post-cisplatin or vehicle exposure. Subcutaneous grafting of tissue pieces was performed by an experienced animal technician; four pieces (∼1 mm^3^) were inserted under the dorsal skin of each host adult CD1-nude mouse using a 13-gauge cancer implant needle (Popper and Sons). Two control- and two cisplatin-exposed tissue pieces from the same fetus were placed along either side of midline, four host mice were grafted per fetal sample. Mice were housed in the same location at room temperature of ∼20–25°C with 12 h light/dark cycles. One week post-xenografting, mice received an injection of 100 μl of 20 IU human chorionic gonadotrophin (hCG; Ovitrelle, Merck Serono) three-times per week. At the end of experiment (12 weeks post-grafting), the retrieval of tissue was performed by humane culling of the mice (inhalation of CO_2_ and cervical dislocation) and locating the surviving tissue xenografts under the skin. One mouse was excluded from the analysis due to poor health and premature end of experiment.

### 2.7 Tissue Processing

Tissue pieces were fixed in Bouin’s liquid (Clin-Tech) for 1 h and transferred to 70% ethanol. Samples were embedded in paraffin blocks and cut into 5 μm serial sections. Every 10th section was subjected for H&E staining to assess tissue integrity prior to performing the immunofluorescent protocol. Only tissue samples that met the criteria for healthy morphology (defined tubules, minimal apoptosis and germ cell presence) in pre-culture and vehicle-exposed control tissue were included in the analysis.

### 2.8 Immunofluorescence

Detailed description of the immunofluorescent protocol can be found in Tharmalingam et al. (2020) and details of antibodies and conditions are provided in Table 2.

**TABLE 2:** Key reagents used in immunofluorescent protocol.

Antibody (Cat no)	Dilution (antigen retrieval)	Origin	Blocking agent	Detection	Cell type detected
*Primary antibodies*					
SOX9 (AB5535)	1:10,000 (none)	Rabbit	Normal goat serum/TBS/BSA	Tyramide-FITC	Sertoli cell
CC3 (#9661)	1:100 (0.01 M citrate buffer)	Rabbit	Normal goat serum/TBS/BSA	Tyramide-Cy3	Apoptotic cell
AMH (sc-6886)	1: 1,000 (none)	Goat	Normal chicken serum/TBS/BSA	Tyramide- Cy5	Sertoli cell
*Secondary antibodies*					
Anti-rabbit peroxidase (PI-1000)	1:200	Goat			
Anti-goat peroxidase (sc-2961)	1:200	Chicken			

Briefly, tissue sections were dewaxed in xylene, rehydrated in decreasing concentrations of ethanol, washed in tap water and placed in antigen retrieval solution for heat-induced antigen retrieval (except for SOX9 staining). Endogenous peroxidase was blocked by 15 min immersion in 3% hydrogen peroxide and non-specific sites were blocked by incubating individual tissue sections with blocking agent containing serum (2.5% BSA, 10% normal goat serum (Biosera, United Kingdom) in TBS). All steps were performed at room temperature. Primary antibody solution was prepared by diluting the stock in the blocking solution and applied to individual tissue sections. Slides were incubated overnight at 4°C.

After washing in TBS, tissue sections were incubated with peroxidase-conjugated secondary antibody for 30 min and visualised using aTyramide signal amplification kit (PerkinElmer, Inc.) at 1:50 for 10 min (both steps at room temperature). Where double staining was performed, the antigen retrieval was carried using microwave treatment prior to CC3 primary antibody and detection steps were repeated using a different fluorophore. Counterstaining with Hoechst (Thermo Fisher Scientific) diluted in TBS at 1:2000 was performed to detect nuclei. Tissue sections were mounted with Permafluor (Lab Vision™, Thermo Scientific).

Staining and analysis was performed on two tissue sections (at least 20 serial sections apart) from two replicates for each treatment per fetal or pre-pubertal tissue sample.

### 2.9 Microscopy

Images used for manual cell counting were acquired using LSM 780 confocal microscope (Carl Zeiss Lt). Tiled images of whole tissue sections were captured at ×20 magnification. Settings were defined for each immunofluorescent run by firstly observing the staining in positive and negative controls (and some control-exposed tissue sections) and ensuring the staining was visible and not overexposed in any of the sections.

Images used for semi-automated quantification were captured using Axio Scan Z.1 slide scanner (Carl Zeiss Ltd., Welwyn Garden City, United Kingdom).

### 2.10 Cell Quantification

For manual counting of Sertoli (SOX9^+ve^) and apoptotic (CC3^+ve^) cells per seminiferous area (mm^2^), images of whole tissue sections obtained from confocal imaging were used. Image analysis was performed using Zen 2 (Blue edition) software (Carl Zeiss Ltd.). Number of positively stained cells was obtained by clicking on each individual cell and total seminiferous area (mm^2^) was calculated by drawing around individual cords/tubules and adding values together. All cords/tubules per whole tissue section were included.

For semi-automated protein expression quantification of Sertoli (SOX9^+ve^) and apoptotic (CC3^+ve^) cells and anti-Müllerian hormone (AMH), ImageJ 1.48v software (Fiji) was used. The channels, representative of the fluorophores were split and measurements for the fluorophore of interest was performed. A threshold was set to ensure accurate detection of fluorophore area comparing it with the original image. The percentage fluorophore area was reported in relation to the section or total cord area that was measured by manually drawing around individual seminiferous cords or tubules and summing the areas. The semi-automated quantification was verified with preliminary manual quantification. This method of quantification has been previously established to be directly comparable with manual quantification (Lopes et al., 2016; Smart et al., 2018).

For both manual and semi-automated counting, analysis was performed for each immunofluorescent run separately with the assessor blinded to treatment/tissue details.

### 2.11 Statistics

Cell counts are presented as mean ± SEM. To account for inter-individual sample variation, two-way ANOVA statistical analysis was performed, using GraphPad Prism nine software (La Jolla, CA, United States). Statistical significance was defined as *p* < 0.05.

## 3 Results

### 3.1 Exposure to Cisplatin had no Effect in Sertoli Cell Number in Human Fetal Testicular Tissues

**FIGURE 1:**
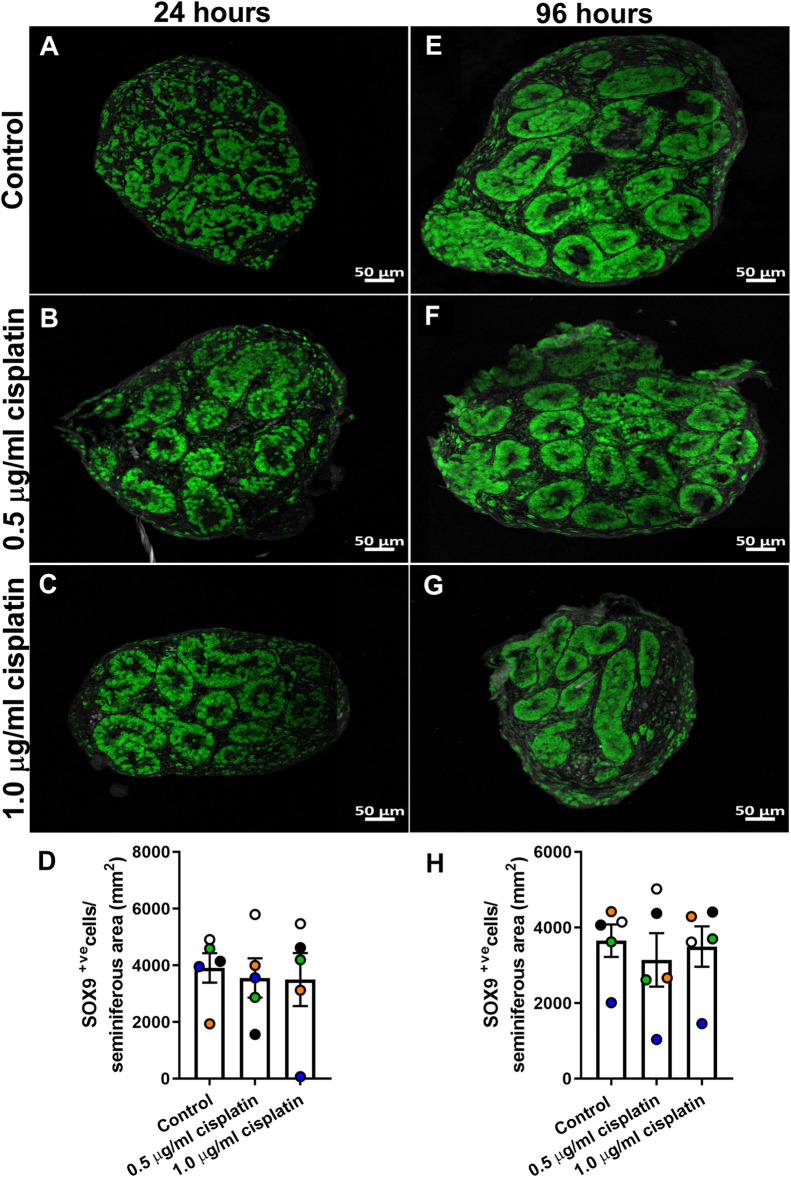
Acute effects of exposure to cisplatin on Sertoli cell numbers in the human fetal testicular tissues. SOX9+ (green) protein expression in the human fetal testicular tissues 24 h **(A–C)** and 96 h **(E–G)** following exposure to vehicle control or cisplatin (0.5 and 1.0 μg/ml). Scale bars represent 50 μm. Quantification of Sertoli cell (SOX^+ve^) numbers per cord area (mm^2^) in the human fetal testicular tissues 24 h **(D)** and 96 h **(H)** following exposure to vehicle control or cisplatin (0.5 and 1.0 μg/ml). Sertoli cell numbers were unaffected at either 24 or 96 h post-exposure to 0.5 μg/ml and 1.0 μg/ml cisplatin. Data analysed using two-way ANOVA. Values shown are means ± SEM and each set of coloured data point represents an individual fetus (*n* = 5).

To determine the acute effects of cisplatin exposure on Sertoli cell number, human fetal testicular tissues (*n* = 5) were exposed to cisplatin (0.5 and 1.0 μg/ml) or vehicle control for 24 h *in vitro* and tissue sections were stained for SOX9 expression at 24 h (Figures 1A–C) and 96 h (Figures 1E–G) post-exposure. Positively stained cells were counted per seminiferous cord area (mm^2^). No change in Sertoli cell numbers were observed at 24 h (3,555 ± 695 (0.5 μg/ml cisplatin) vs. 3,494 ± 936 (1.0 μg/ml cisplatin) vs. 3,905 ± 520 (control) cells/mm^2^, *p* ≥ 0.05; Figure 1D) or 96 h (3,142 ± 706 (0.5 μg/ml cisplatin) vs. 3,494 ± 533 (1.0 μg/ml cisplatin) vs. 3,650 ± 430 (control) cells/mm^2^, *p* > 0.05; Figure 1H) post-exposure to cisplatin at both concentrations, compared to control.

**FIGURE 2:**
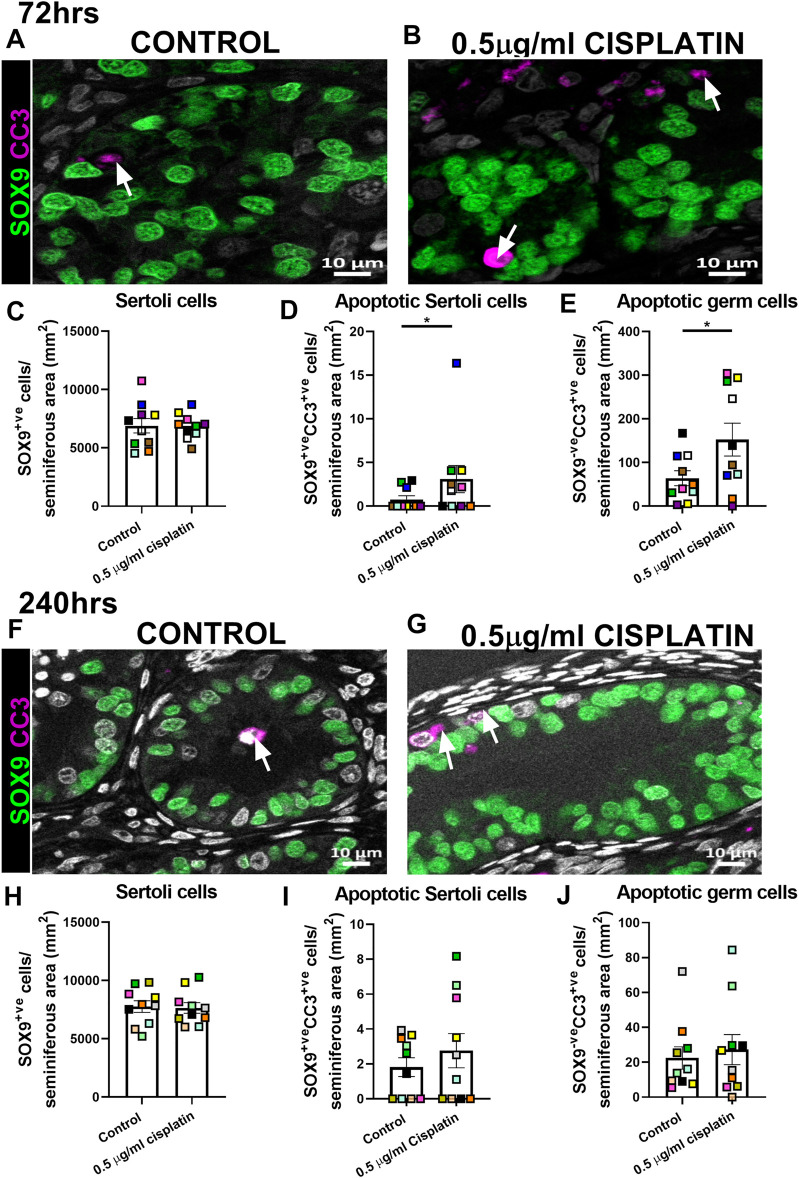
Intermediate and prolonged effects of exposure to cisplatin on Sertoli cell number and apoptosis within cords in the human fetal testicular tissues. SOX9 (green) and cleaved caspase 3 (CC3; purple) protein expression in the human fetal testicular tissues 72 h **(A,B)** and 240 h **(F,G)** following exposure to vehicle control or cisplatin (0.5 μg/ml). Scale bars represent 10 μm, arrows point to apoptotic germ cells (SOX9^−ve^CC3^+ve^). Quantification of Sertoli cell (SOX9^+ve^; C&H), apoptotic Sertoli cell (SOX9^+ve^CC3^+ve^; D&I) and apoptotic germ cell (SOX9^−ve^CC3^+ve^; E&J) numbers per cord area (mm^2^) in the human fetal testicular tissues 72 h **(C–E)** and 240 h **(H–J)** following exposure to vehicle control or cisplatin (0.5 μg/ml). Sertoli cell numbers were unaffected at either 72 or 240 h post-exposure to 0.5 μg/ml cisplatin. Significant increase in apoptotic Sertoli and apoptotic germ cell numbers was observed at 72 h but not 240 h post-exposure. Data analysed using two-way ANOVA (*p < 0.05). Values shown are means ± SEM and each set of coloured data points represents an individual fetus (*n* = 10).

Given that no acute change in Sertoli cell number was observed, we aimed to determine whether exposure to cisplatin (0.5 μg/ml) affected the Sertoli cell population in human fetal testicular tissues (*n* = 10) at intermediate (72 h) and prolonged (240 h) post-exposure time-points (Figure 2). Quantification of SOX9^+ve^ cells per seminiferous cord area (mm^2^) revealed no change in Sertoli cell number in cisplatin-exposed tissues compared to control at either 72 h (6,863 ± 343 vs. 6,900 ± 622 cells/mm^2^, *p* > 0.05; Figures 2A–C) or 240 h (7,647 ± 459 vs. 7,767 ± 498 cells/mm^2^, *p* > 0.05; Figures 2F–H) post-exposure. Apoptotic Sertoli cells (SOX9^+ve^/CC3^+ve^ cells; Figures 2D,I) and apoptotic germ cells (SOX9^−ve^CC3^+ve^ cells; Figure 2E,J) were very rare at both time-points and most of the apoptotic cells were SOX9^−ve^, indicative of germ cells. At 72 h post-exposure, the number of apoptotic Sertoli cells (3 ± 2 vs. 1 ± 0 cells/mm^2^, *p* < 0.05; Figure 2D) and apoptotic germ cell (152 ± 38 vs. 64 ± 17 cells/mm^2^, *p* < 0.05; Figure 2E) were significantly increased in cisplatin-exposed tissues compared to control; however, it is important to note that the increased number of apoptotic Sertoli cells appeared to be the result of an outlier in the cisplatin group (Figure 2D, blue square). At 240 h post-exposure, no difference in the number of apoptotic Sertoli cells (3 ± 1 vs. 2 ± 1 cells/seminiferous area (mm^2^), *p* > 0.05; Figure 2I) or apoptotic germ cells (27 ± 9 vs. 22 ± 6 cells/seminiferous area (mm^2^), *p* > 0.05; Figure 2J) was observed.

### 3.2 Exposure to Cisplatin Did Not Affect Sertoli Cell Function in Human Fetal Testicular Tissues

Exposure to cisplatin did not affect Sertoli cell function in human fetal testicular tissues.

Given that Sertoli cell number, except the apoptotic Sertoli cell number, was unaffected by cisplatin exposure at all time-points, we investigated whether exposure to cisplatin (0.5 and 1.0 μg/ml) resulted in effects on Sertoli cell maturation and function through quantification of AMH expression (immature Sertoli cell marker) and measurement of hormone production (AMH and inhibin B), respectively.

**FIGURE 3:**
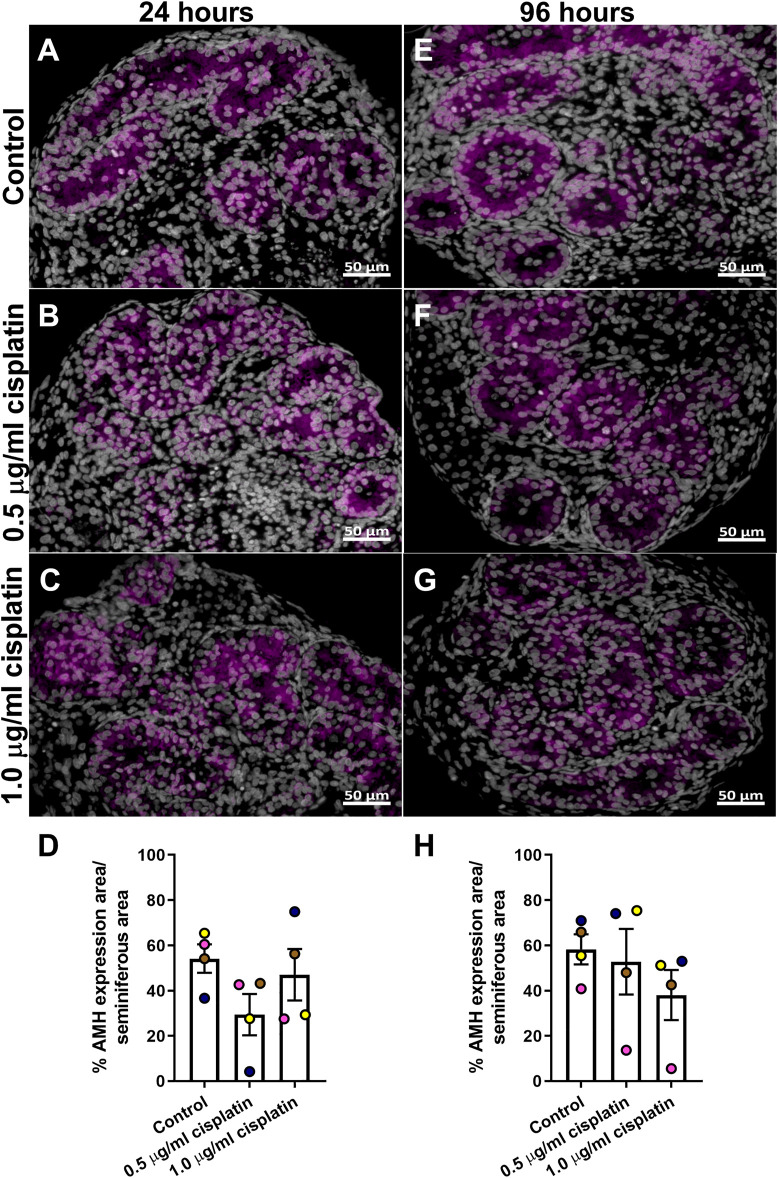
Acute effects of exposure to cisplatin on Sertoli cell maturation in the human fetal testicular tissues. Anti-Mϋllerian hormone (AMH; purple) protein expression in the human fetal testicular tissues exposed to vehicle control **(A,E)**, 0.5 μg/ml cisplatin **(B,F)** or 1.0 μg/ml cisplatin **(C,G)** for 24 h and further cultured until 24 **(A–C)** and 96 **(E–G)** hrs post-exposure. Scale bars represent 50 μm. Quantification of percentage of cord area expressing AMH **(D,H)**. Exposure to cisplatin did not have an acute effect on the percentage of cord area expressing AMH. Data analysed using two-way ANOVA. Values shown are means ± SEM and each of coloured data points represents an individual fetus (*n* = 4).

Tissue sections were stained for AMH (Figures 3A–C,E–G) and quantification revealed that percentage of AMH expression per seminiferous cord area was not different in the cisplatin-exposed (0.5 or 1.0 μg/ml) compared to vehicle-exposed testicular tissue at either 24 h (29 ± 9 (0.5 μg/ml cisplatin) vs. 47 ± 11 (1.0 μg/ml cisplatin) vs. 54 ± 6 (control) %, *p* > 0.05; Figure 3D) and 96 h (53 ± 14 (0.5 μg/ml cisplatin) vs. 38 ± 11 (1.0 μg/ml cisplatin) vs. 58 ± 7 (control) %, *p* > 0.05; Figure 3H) post-exposure.

**FIGURE 4:**
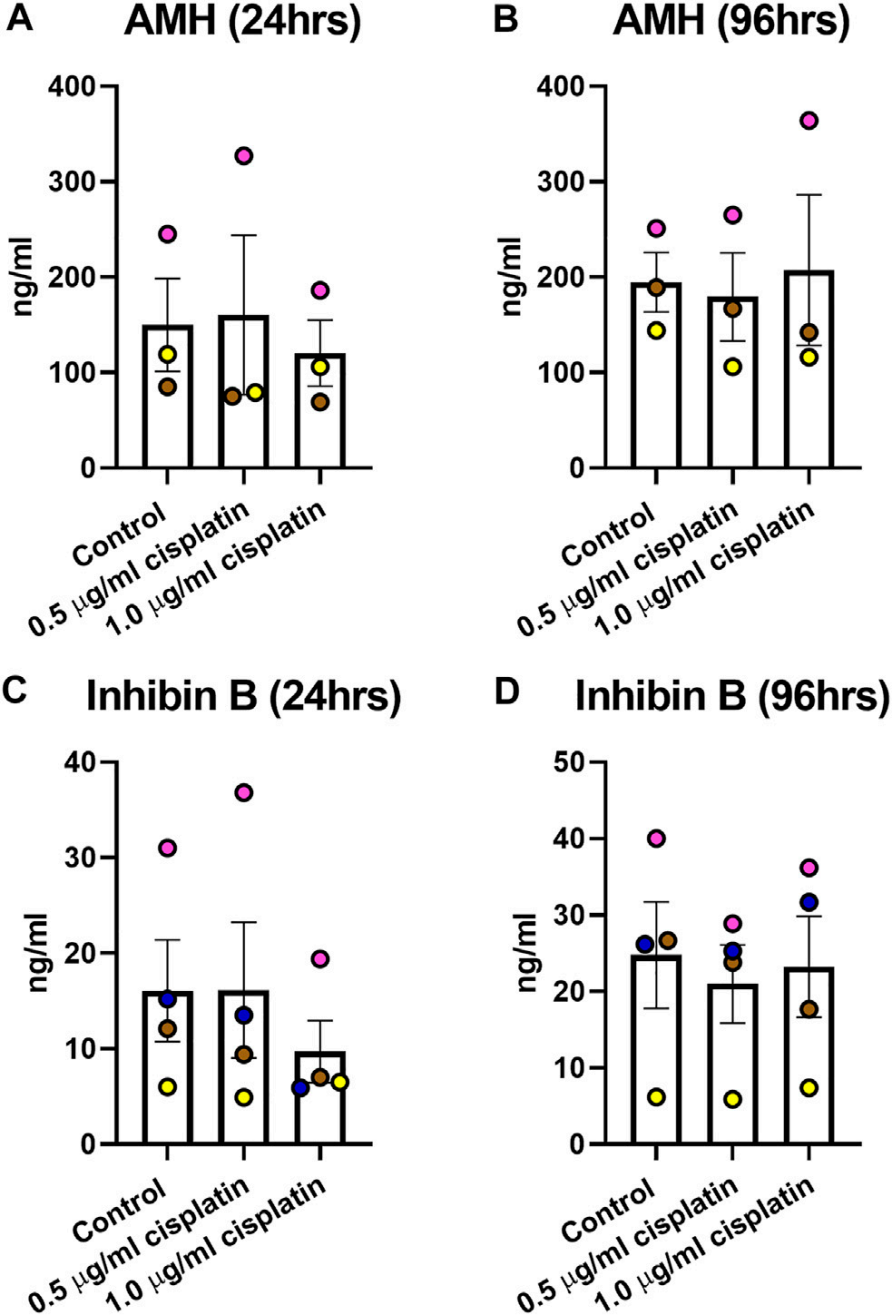
Acute effects of exposure to cisplatin on human fetal testicular function. Measurement of levels of inhibin B **(A,B)** and anti-Mϋllerian hormone [AMH; **(C,D)** ]in culture medium of human fetal testicular tissues at 24 and 96 h post-exposure. No differences in the levels of inhibin B and AMH were detected at 24 or 96 h post-exposure to 0.5 μg/ml and 1.0 μg/ml cisplatin. Data analysed using two-way ANOVA. Values shown are means ± SEM and each set of coloured data point represents an individual fetus (*n* = 3–4).

Culture medium was collected to determine the levels of AMH and inhibin B (Figure 4). AMH production was unaffected by exposure to cisplatin at 24 h (160 ± 83 (0.5 μg/ml cisplatin) vs. 120 ± 35 (1.0 μg/ml cisplatin) vs. 150 ± 49 (control) ng/ml, *p* > 0.05; Figures 4A ad 96 h (179 ± 46 (0.5 μg/ml cisplatin) vs. 207 ± 79 (1.0 μg/ml cisplatin) vs. 195 ± 31 (control) ng/ml, *p* > 0.05; Figure 4B) post-exposure. Similarly, there was no effect of cisplatin exposure on inhibin B at 24 h (16 ± 7 (0.5 μg/ml cisplatin) vs. 10 ± 3 (1.0 μg/ml cisplatin) vs. 16 ± 5 (control) ng/ml, *p* > 0.05; Figures 4C and 96 h (21 ± 5 (0.5 μg/ml cisplatin) vs. 23 ± 7 (1.0 μg/ml cisplatin) vs. 25 ± 7 (control) ng/ml, *p* > 0.05; Figure 4D) post-exposure.

### 3.3 Exposure to Cisplatin had no Long-Term Effects on Sertoli Cell Number and Function in Human Fetal Testicular Xenografts

**FIGURE 5:**
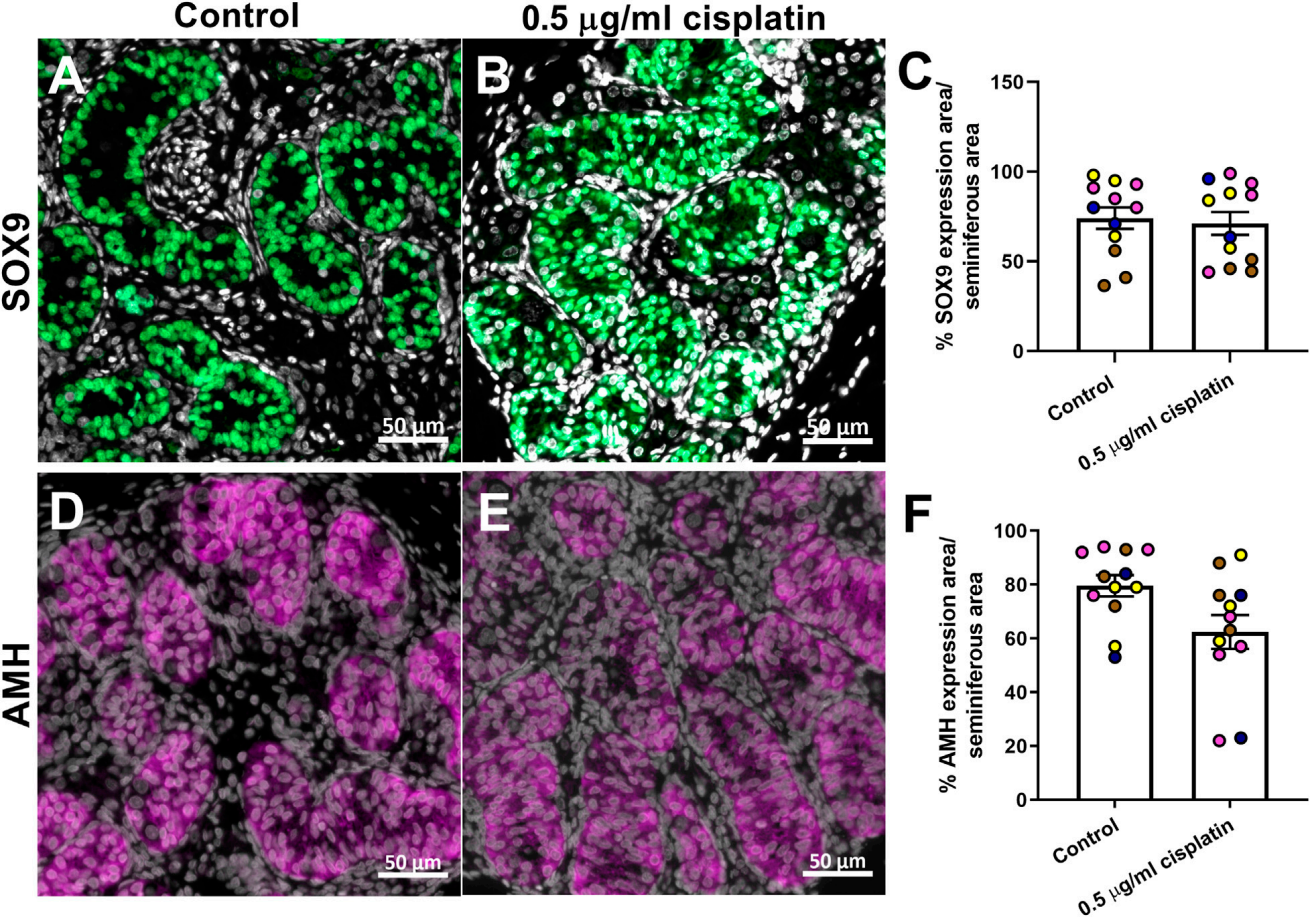
Long-term effects of exposure to cisplatin on Sertoli cells in the human fetal testicular tissue xenografts. SOX9 [green; **(A,B)**] and anti-Mϋllerian hormone [AMH; purple, **(D,E)**] protein expression in the human fetal testicular tissues exposed to vehicle control or 0.5 μg/ml cisplatin for 24 h prior to xenografting (12 weeks). Scale bars represent 50 μm. Quantification of percentage of cord area expressing SOX9 **(C)** or AMH **(F)** in xenografts. Exposure to 0.5 μg/ml cisplatin did not have a long-term effect on the percentage of cord area expressing SOX9 or AMH. Data analysed using two-way ANOVA. Values shown are means ± SEM and each of coloured data points represents an individual fetus (*n* = 4).

To evaluate long-term impact of cisplatin exposure on Sertoli cells, sections of human fetal testicular tissue that were exposed *in vitro* and xenografted for a further 12 weeks were stained for SOX9 expression (Figures 5A,B). Quantification of percentage of seminiferous cord area that was positively-stained for SOX9 revealed no difference between cisplatin- and control-exposed tissues (71 ± 6 vs. 74 ± 6%, *p* > 0.05; Figure 5C). To understand whether function of Sertoli cells was impacted by exposure to cisplatin, immunohistochemistry was performed for AMH (immature Sertoli cell marker) expression (Figures 5D,E). Quantification of percentage of seminiferous area expressing AMH showed no change in AMH expression between cisplatin- and control-exposed tissues (62 ± 6 vs. 80 ± 4%, *p* > 0.05; Figure 5F).

### 3.4 No Effect on Sertoli Cell Number and Function in Human Prepubertal Testicular Tissues Exposed to Platinum-Based Chemotherapeutic Agents

**FIGURE 6:**
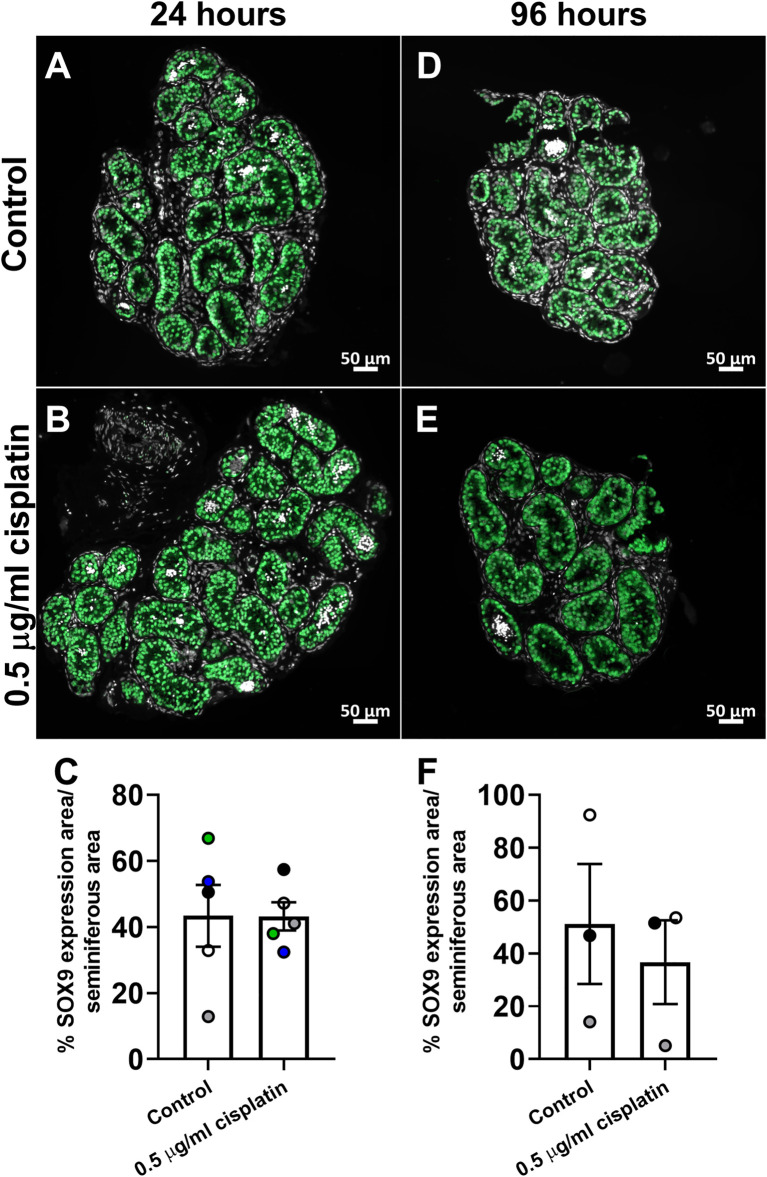
Effects of exposure to cisplatin on Sertoli cells in human pre-pubertal testicular tissues. SOX9 (green) protein expression in the human pre-pubertal testicular tissues exposed to vehicle control **(A,D)** or 0.5 μg/ml cisplatin **(B,E)** for 24 h and further cultured until 24 **(A,B)** and 96 **(D,E)** hrs post-exposure. Scale bars represent 50 μm. Quantification of percentage of tubular area expressing SOX9 **(C)** in human pre-pubertal testicular tissues at 24 h **(C)** and 96 h **(F)** post-exposure. No effect was observed on the percentage of tubular area expressing SOX9 in human pre-pubertal testicular tissues in tissues exposed to cisplatin compared to control. Data analysed using two-way ANOVA. Values shown are means ± SEM and each set of coloured data points (white and black—1 year, green—8 years, blue—11 years and grey—12 years) represents an individual patient (*n* = 3–5).

In order to translate these findings to the childhood testis, we exposed human pre-pubertal testicular tissues to cisplatin using the same *in vitro* approach described for the human fetal testis tissues. Sections were stained for SOX9 expression and percentage of seminiferous area that expressed SOX9 was quantified (Figures 6A–F). No differences in area of SOX9 expression between cisplatin- or control-exposed tissues were observed at either 24 h (43 ± 4 vs. 43 ± 9%, *p* > 0.05; Figure 6C) or 96 h (37 ± 16 vs. 51 ± 23%, *p* > 0.05; Figure 6F).

The function of Sertoli cells to maintain an immature niche in human pre-pubertal testis tissue was investigated through AMH expression post cisplatin exposure (Supplementary Figure S1). At 24 h post-exposure, there was a significant decrease in AMH expression area in cords post cisplatin exposure compared to vehicle controls (9 ± 2 vs. 22 ± 10%, *p* < 0.05; Supplementary Figure S1C). At 96 h post-exposure, the percentage AMH expression in cords was similar (50 ± 22 vs. 57 ± 22s%, *p* > 0.05; Supplementary Figure S1F).

**FIGURE 7:**
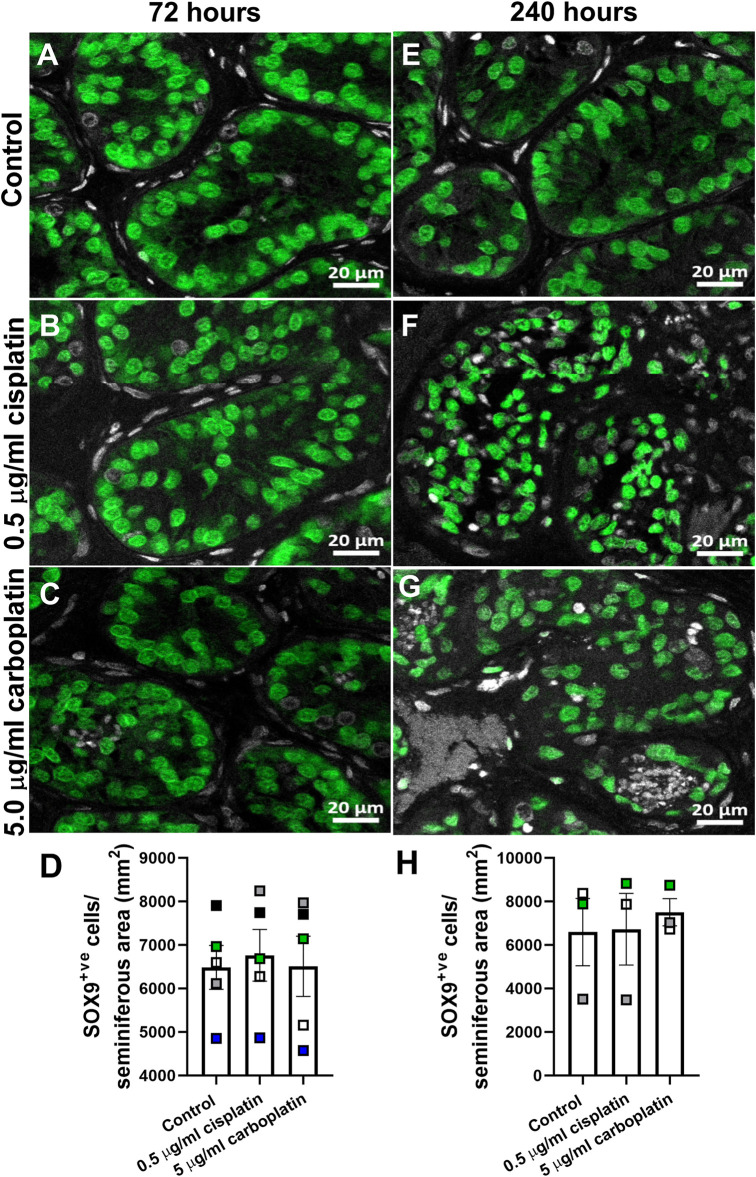
Effects of exposure to cisplatin and carboplatin on Sertoli cell number in the human pre-pubertal testicular tissues. SOX9 (green) protein expression in the human pre-pubertal testicular tissues 72 h **(A–C)** and 240 h **(E–G)** following exposure to vehicle control, cisplatin (0.5 μg/ml) or carboplatin (5 μg/ml). Scale bars represent 20 μm. Quantification of Sertoli cell (SOX9^+ve^) numbers per tubular area (mm^2^) in the human pre-pubertal testicular tissues 72 h **(D)** and 240 h **(H)** following exposure to vehicle control, cisplatin (0.5 μg/ml) or carboplatin (5 μg/ml). Exposure to cisplatin or carboplatin had no effect on the Sertoli cell numbers in human pre-pubertal testicular tissues at either 72 or 240 h post-exposure Data analysed using two-way ANOVA. Data analysed using two-way ANOVA. Values shown are means ± SEM and each set of coloured data points (black—2 years, grey and green—4 years, white—7 years and blue—8 years old) represents an individual patient (*n* = 3–5).

To compare the effects of exposure to cisplatin and carboplatin on Sertoli cell number in human pre-pubertal testicular tissues, sections were stained for SOX9 and positively-stained cells counted per tubular area (mm^2^) (Figures 7A–H). No difference in Sertoli cell number was observed in cisplatin- or carboplatin-exposed tissues compared to control at either 72 h (6,762 ± 592 vs. 6,510 ± 691 vs. 6,486 ± 504 cells/mm^2^, *p* ≥ 0.05; Figure 7D) or 240 h (6,723 ± 1,647 vs. 7,502 ± 627 vs. 6,592 ± 1,545 cells/mm^2^, *p* ≥ 0.05; Figure 7H) post-exposure.

## 4 Discussion

The findings of this experimental study demonstrate that Sertoli cell number, function and maturation are preserved in cisplatin- or carboplatin-exposed immature human testicular tissues.

### 4.1 Maintenance Sertoli Cell Number in the Immature Human Testis Exposed to Platinum-Based Chemotherapy

It is known that Sertoli cell number in adulthood determines the number of germ cells that can be supported, hence the amount of sperm that can be produced (Sharpe et al. (2003). Sertoli cell proliferation in primates occur during two distinct developmental periods, including a fetal/neonatal and a peri-pubertal phase (Cortes et al., 1987; Marshall and Plant, 1996; Sharpe et al., 2000). Once adulthood is reached, Sertoli cells are fully mature and cannot proliferate to replace any lost Sertoli cells (Sharpe et al., 2000) This contrasts to the situation in rodents, where Sertoli cell proliferation occurs throughout development until the initiation of puberty (Orth, 1982; Vergouwen et al., 1991; Sharpe et al., 2000). Given this, maintaining Sertoli cell number during pre-puberty in the primate testis is essential to preserve fertility potential. In this study, we have shown that Sertoli cell number is maintained in human fetal and pre-pubertal testicular tissues exposed to cisplatin, compared to control. This is despite the loss of germ cells that occurs with exposure to platinum-based chemotherapy (Tharmalingam et al., 2020). As such, it can be postulated that fertility-related issues in adult childhood cancer survivors do not result from a reduction Sertoli cell number. Taken together with our previous findings showing that spermatogonial numbers were reduced in immature human testicular tissue after cisplatin exposure (Tharmalingam et al., 2020), it could be proposed that exposure to cisplatin induces a direct damage to the germ cells, resulting in reduced fertility potential, however, the other cell types within the testicular tissue (e.g. Leydig cells, peritubular myoid cells) have not been explored yet.

### 4.2 Sertoli Cell Hormone Production as an Indicator of Sertoli Cell Function Post-Chemotherapy Exposure

Whilst Sertoli cell number remains unaffected by exposure to platinum-based chemotherapy, it is important to also assess Sertoli cell functionality to maintain and support germ cell development. Sertoli cell function in the fetal and prepubertal human testis can be determined by measurement of two key hormones; inhibin B and AMH. AMH is produced during fetal and pre-pubertal life with a dramatic reduction in secretion during puberty, coinciding with increase in testosterone production (Aksglaede et al., 2010). Therefore, a reduction in AMH expression or secretion can be indicative of either loss of function or maturation of Sertoli cells. Our results show that AMH production is maintained in the human fetal and pre-pubertal testis after exposure to cisplatin. Although an initial decrease in AMH is observed in the pre-pubertal testis, AMH expression recovered and was similar to the control. The findings suggest that function is maintained in Sertoli cells after cisplatin exposure, with no premature maturation towards terminal differentiation, thus keeping the testis in immature state similar to that observed in the controls.

The present study also found no effect of cisplatin exposure on inhibin B production from immature human testis. Inhibin B can be used as an indicator of Sertoli cell function in males from fetal life until adulthood. Serum levels of inhibin B peak in infancy during first 2 month of life. This is followed by another peak in puberty, which is maintained into adulthood (Kelsey et al., 2016). Inhibin B levels in plasma are used to assess effects on Sertoli cell function induced by chemotherapy treatment in adults following childhood cancer treatment (Thomson et al., 2002; Utriainen et al., 2019). In a study involving 33 adult survivors of childhood cancer and 66 age-matched controls, 10 patients had undetectable levels of inhibin B, whereas all controls had normal levels of inhibin B (Thomson et al., 2002). Another study involving nine males treated with a combination of alkylating agents and cisplatin showed a significantly reduced inhibin B serum levels in the treated patients (0–26 vs. 138–173 ng/L; *p* < 0.001), compared with nine control males (Utriainen et al., 2019). These studies support the evaluation of inhibin B as an indicator of Sertoli cell function and impaired spermatogenesis. Thus suggesting that alkylating chemotherapy drugs are associated with reduced inhibin B production and impaired Sertoli cell function (Thomson et al., 2002).

Inhibin B levels in plasma may also be measured in childhood to provide an early indicator of Sertoli cell damage induced by chemotherapy treatment (Crofton et al., 2003). Levels of inhibin B in serum were analysed in boys immediately prior to chemotherapy treatment and 1–2 weeks after treatment was complete. No change in inhibin B levels were detected in the majority of patients (Crofton et al., 2003). However, the one patient who received treatment that included cisplatin had no detectable inhibin B after the completion of treatment, suggesting impaired Sertoli cell function (Crofton et al., 2003). Our results suggest that if inhibin B was reduced as a result of chemotherapy exposure, it would have been due to the alkylating agents received (cyclophosphamide and melphalan), rather than the cisplatin treatment.

In adulthood, Sertoli cells support spermatogenesis by secreting various growth factors, such as Insulin-like growth factor-I (IGF-I), fibroblast growth factor (FGF), interleukin-1*α* (IL-1*α*), transforming growth factor-*α*/*β* (TGF-*α*/*β*) and stem cell factor (SCF), that are believed to be essential for both supporting pre- and post-meiotic germ cells (reviewed in Petersen and Soder (2006)). Furthermore, growth factors may also act as ligands for receptors present on the SSCs, directly affecting SSC development. For example, GDNF is secreted by Sertoli cells and PTMs and has been shown to bind to SSCs that express, GFRα1 (GDNF receptor), in human adult testes (Singh et al., 2017). Based on rodent studies, GDNF has been shown to play a role in balancing self-renewal and differentiation of SSCs depending on the levels of GDNF present (reviewed in Hofmann (2008)). CXCL12, also secreted by Sertoli cells, acts on CXCR4 receptors present on spermatogonia, including a sub-population of SSCs, in mouse testes and has been shown to inhibit differentiation of SSCs (Yang et al., 2013). Therefore, whilst the present results of hormone analysis indicate that Sertoli cell function is maintained, further investigation into the Sertoli cell proteome and secretome profile in chemotherapy-exposed testes would provide additional information on whether Sertoli cell signalling pathways that are important in maintaining and regulating the SSC niche are retained in the chemotherapy-exposed testes.

### 4.3 The Human Fetal Testis as a Model to Investigate Effects of Chemotherapy Exposure in Prepubertal Testis

Previously we proposed the utilisation of human fetal testicular tissues as a model to study effects of exposure to pharmaceutical compounds in pre-pubertal testis (Tharmalingam et al., 2020). In experiments using both human fetal and pre-pubertal testicular tissues, no effect on germ cell numbers was observed at 24 h post-exposure whereas significant reduction in (pre)spermatogonial cell number was seen at 96 h post-exposure. In the current study we show that preserved Sertoli cell numbers and functionality were observed in cisplatin- and carboplatin-exposed human fetal and pre-pubertal testicular tissues at all time-points. This finding further supports the use of human fetal testicular tissues as a surrogate model for pre-pubertal testis where tissue availability is limited.

Moreover, it could be proposed that exposure of the immature human testicular tissues to cisplatin could be used as a model to study the strategies for fertility preservation, such as SSC transplantation, mesenchymal stem cell injection or treatment with exogenous chemoprotective agents. The gold standard treatment to establish the infertility model is treatment with busulfan which completely depletes the testes of germ cells (Brinster and Zimmermann, 1994). Although busulfan and cisplatin are generally categorised into the same chemotherapeutic drug group–alkylating agents–busulfan is an alkyl alkane sulphonate and cisplatin is an alkylating-like platinum-based compound (Lind, 2011). Therefore, it might be essential to establish study models for each individual drug due to slight differences in mechanisms of action. Having such models would provide a more suitable tool to investigate chemoprotective strategies for chemotherapeutic drugs. It is postulated that ‘one fit for all’ approach might not be applicable when exploring the chemoprotective candidates as certain agents might reduce the side effects with one chemotherapeutic agent but exaggerate the side effects with another drug.

### 4.4 Importance of Maintaining Sertoli Cell Number and Function After Chemotherapy Exposure for Fertility Preservation in Prepubertal Boys

Maintaining normal Sertoli cell number and function is important when considering the future effectiveness of fertility preservation strategies for pre-pubertal boys with cancer. Testicular tissue can be obtained from boys prior to cancer treatment and cryopreserved for use in future fertility restoration (Onofre et al., 2016). Expansion of SSCs from cryopreserved tissues *in vitro* followed by re-transplantation into the seminiferous tubules testis in adulthood represents an approach to restoring natural fertility and has been successfully applied to producing offspring in mice (Brinster and Zimmermann, 1994) and for generating functional sperm in primates (Hermann et al., 2012; Shetty et al., 2020). The success of SSC expansion *in vitro* has been shown to be dependent on preserved somatic cell characteristics (Gat et al., 2017). SSC and somatic cell suspensions were obtained from adult testicular tissue and cultured for 12 days separately and together. It showed that co-culturing germ cells with a small number of testicular somatic cells compared to germ cells-only resulted in 2-times higher number of germ cell aggregates formed (Gat et al., 2017), showing the importance of Sertoli cells in expansion of SSCs.

The role of the somatic cells in supporting transplanted SSC colonization and differentiation has also been shown in studies involving testicular irradiation treatment (Zhang et al., 2007). Transplantation of SSCs from untreated prepubertal rats into testes of irradiated rats resulted in colonization of SSCs in the seminiferous tubules but these spermatogonia did not differentiate, indicating that injury to the somatic cells can prevent successful restoration of spermatogenesis with SSC transplantation (Zhang et al., 2007). Maintenance of Sertoli cell number is also likely to be important for successful SSC transplantation as this has been shown to be a key regulator in establishing SSC niches post-transplantation (Oatley et al., 2011). SSC transplantation was performed to the testes of adult mice treated with polythiouracil (PTU), which is known to extend the period of Sertoli cell proliferation thus resulting in increased Sertoli cell number (Oatley et al., 2011). Compared to transplantation into untreated mice, a three-fold increase in the number of SSC niches was observed in the transplanted testes from PTU-treated mice, compared with control (Oatley et al., 2011). These results provided direct evidence that the success of SSC transplantation was dependent on Sertoli cell content in recipient testes.

## 5 Conclusion

Our current study shows that Sertoli cell number and function were preserved in immature human testicular tissues exposed to platinum-based chemotherapeutic agents. Knowing that Sertoli cell number and function are important in effectiveness of fertility preservation strategies, this indicates that if a pre-pubertal boy was to receive platinum-based chemotherapy treatment followed by SSC transplantation, these Sertoli cells would potentially be capable of supporting the establishment of SSC niches.

## Data Availability

The raw data supporting the conclusion of this article will be made available by the authors, without undue reservation.

## Supplementary Material

Supplementary Figure S1 Acute effects of exposure to cisplatin on Sertoli cell maturation in the human pre-pubertal testicular tissues. Anti-Müllerian hormone (AMH; purple) protein expression in the human pre-pubertal testicular tissues exposed to vehicle control **(A, D)** or 0.5 μg/ml cisplatin **(B, E)** for 24 hrs and further cultured until 24 **(A–B)** and 96 **(D–E)** hrs post-exposure. Scale bars represent 50 μm. Quantification of percentage of cord area expressing AMH. Exposure to 0.5 μg/ml cisplatin did not have an acute effect on the percentage of cord area expressing AMH. Data analysed using two-way ANOVA. Values shown are means ± SEM and each of coloured data points represents an individual sample (*n* = 3–4).

## References

[B1] Aksglaede L. Sørensen K. Boas M. Mouritsen A. Hagen C. P. Jensen R. B. (2010). Changes in Anti-müllerian Hormone (AMH) throughout the Life Span: A Population-Based Study of 1027 Healthy Males from Birth (Cord Blood) to the Age of 69 Years. J. Clin. Endocrinol. Metab. 95, 5357–5364. 10.1210/jc.2010-1207 20843948

[B2] Allen C. M. Mitchell R. T. Spears N. Spears N. (2018). How Does Chemotherapy Treatment Damage the Prepubertal Testis? Reproduction 156, R209–R233. 10.1530/rep-18-0221 30394705 PMC6347281

[B3] Anderson R. A. Kelsey T. W. Telfer E. E. (2015). Cancer Treatment and Gonadal Function: Experimental and Established Strategies for Fertility Preservation in Children and Young Adults. Lancet Diabetes Endocrinol. 3, 556–567. 10.1016/s2213-8587(15)00039-x 25873571

[B4] Anderson R. A. Wallace E. M. Groome N. P. Bellis A. J. Wu F. C. (1997). Physiological Relationships between Inhibin B, Follicle Stimulating Hormone Secretion and Spermatogenesis in normal Men and Response to Gonadotrophin Suppression by Exogenous Testosterone. Hum. Reprod. 12, 746–751. 10.1093/humrep/12.4.746 9159436

[B5] Anderson R. A. Wallace W. H. B. (2016). Chemotherapy Risks to Fertility of Childhood Cancer Survivors. Lancet Oncol. 17, 540–541. 10.1016/s1470-2045(16)00116-9 27020006

[B6] Andersson A.-M. Skakkebæk N. E. (2001). Serum Inhibin B Levels during Male Childhood and Puberty. Mol. Cell Endocrinol. 180, 103–107. 10.1016/s0303-7207(01)00520-2 11451578

[B7] Bergadá I. Milani C. Bedecarrás P. Andreone L. Ropelato M. G. Gottlieb S. (2006). Time Course of the Serum Gonadotropin Surge, Inhibins, and Anti-müllerian Hormone in Normal Newborn Males during the First Month of Life. J. Clin. Endocrinol. Metab. 91, 4092–4098. 10.1210/jc.2006-1079 16849404

[B8] Brinster R. L. Zimmermann J. W. (1994). Spermatogenesis Following Male Germ-Cell Transplantation. Proc. Natl. Acad. Sci. 91, 11298–11302. 10.1073/pnas.91.24.11298 7972053 PMC45218

[B9] Chemes H. E. (2001). Infancy Is Not a Quiescent Period of Testicular Development. Int. J. Androl. 24, 2–7. 10.1046/j.1365-2605.2001.00260.x 11168644

[B10] Chow E. J. Stratton K. L. Leisenring W. M. Oeffinger K. C. Sklar C. A. Donaldson S. S. (2016). Pregnancy after Chemotherapy in Male and Female Survivors of Childhood Cancer Treated between 1970 and 1999: a Report from the Childhood Cancer Survivor Study Cohort. Lancet Oncol. 17, 567–576. 10.1016/s1470-2045(16)00086-3 27020005 PMC4907859

[B11] Cortes D. Müller J. Skakkebæk N. E. (1987). Proliferation of Sertoli Cells during Development of the Human Testis Assessed by Stereological Methods. Int. J. Androl. 10, 589–596. 10.1111/j.1365-2605.1987.tb00358.x 3654012

[B12] Crofton P. M. Thomson A. B. Evans A. E. M. Groome N. P. Bath L. E. Kelnar C. J. H. (2003). Is Inhibin B a Potential Marker of Gonadotoxicity in Prepubertal Children Treated for Cancer? Clin. Endocrinol. (Oxf) 58, 296–301. 10.1046/j.1365-2265.2003.01712.x 12608934

[B13] Dasari S. Bernard Tchounwou P. (2014). Cisplatin in Cancer Therapy: Molecular Mechanisms of Action. Eur. J. Pharmacol. 740, 364–378. 10.1016/j.ejphar.2014.07.025 25058905 PMC4146684

[B14] Gat I. Maghen L. Filice M. Wyse B. Zohni K. Jarvi K. (2017). Optimal Culture Conditions Are Critical for Efficient Expansion of Human Testicular Somatic and Germ Cells *In Vitro* . Fertil. Sterility 107, 595–605. 10.1016/j.fertnstert.2016.12.028 28259258

[B15] Goodsell D. S. (2006). The Molecular Perspective: Cisplatin. Stem Cells 24, 514–515. 10.1634/stemcells.2006-csc2 16582013

[B16] Green D. M. Liu W. Kutteh W. H. Ke R. W. Shelton K. C. Sklar C. A. (2014a). Cumulative Alkylating Agent Exposure and Semen Parameters in Adult Survivors of Childhood Cancer: a Report from the St Jude Lifetime Cohort Study. Lancet Oncol. 15, 1215–1223. 10.1016/s1470-2045(14)70408-5 25239573 PMC4192599

[B17] Green D. M. Nolan V. G. Goodman P. J. Whitton J. A. Srivastava D. Leisenring W. M. (2014b). The Cyclophosphamide Equivalent Dose as an Approach for Quantifying Alkylating Agent Exposure: a Report from the Childhood Cancer Survivor Study. Pediatr. Blood Cancer 61, 53–67. 10.1002/pbc.24679 23940101 PMC3933293

[B18] Hermann B. P. Sukhwani M. Winkler F. Pascarella J. N. Peters K. A. Sheng Y. (2012). Spermatogonial Stem Cell Transplantation into Rhesus Testes Regenerates Spermatogenesis Producing Functional Sperm. Cell Stem Cell 11, 715–726. 10.1016/j.stem.2012.07.017 23122294 PMC3580057

[B19] Hofmann M.-C. (2008). Gdnf Signaling Pathways within the Mammalian Spermatogonial Stem Cell Niche. Mol. Cell Endocrinol. 288, 95–103. 10.1016/j.mce.2008.04.012 18485583 PMC2491722

[B20] Hutka M. Smith L. B. Wallace W. H. B. Stukenborg J. B. Mitchell R. T. (2020). Exogenous Gonadotrophin Stimulation Induces Partial Maturation of Human Sertoli Cells in a Testicular Xenotransplantation Model for Fertility Preservation. J. Clin. Med. 9. 10.3390/jcm9010266 PMC7019512 31963729

[B21] Jørgensen A. Young J. Nielsen J. E. Joensen U. N. Toft B. G. Rajpert-De Meyts E. (2014). Hanging Drop Cultures of Human Testis and Testis Cancer Samples: a Model Used to Investigate Activin Treatment Effects in a Preserved Niche. Br. J. Cancer 110, 2604–2614. 10.1038/bjc.2014.160 24781282 PMC4021512

[B22] Kelsey T. W. Miles A. Mitchell R. T. Anderson R. A. Wallace W. H. B. (2016). A Normative Model of Serum Inhibin B in Young Males. PLoS One11 11, e0153843. 10.1371/journal.pone.0153843 PMC4831823 27077369

[B23] Lind M. J. (2011). Principles of Cytotoxic Chemotherapy. Medicine 39, 711–716. 10.1016/j.mpmed.2011.09.009

[B24] Lopes F. Smith R. Nash S. Mitchell R. T. Spears N. (2016). Irinotecan Metabolite SN38 Results in Germ Cell Loss in the Testis but Not in the Ovary of Prepubertal Mice. Mol. Hum. Reprod. 22, 745–755. 10.1093/molehr/gaw051 27470502 PMC5099998

[B25] Malhotra V. Perry M. C. (2003). Classical Chemotherapy: Mechanisms, Toxicities and the Therapeutic Window. Cancer Biol. Ther. 2, S2–S4. 10.4161/cbt.199 14508075

[B26] Marshall G. R. Plant T. M. (1996). Puberty Occurring Either Spontaneously or Induced Precociously in Rhesus Monkey (Macaca Mulatta) Is Associated with a Marked Proliferation of Sertoli Cells1. Biol. Reprod. 54, 1192–1199. 10.1095/biolreprod54.6.1192 8724345

[B27] Oatley M. J. Racicot K. E. Oatley J. M. (2011). Sertoli Cells Dictate Spermatogonial Stem Cell Niches in the Mouse Testis. Biol. Reprod. 84, 639–645. 10.1095/biolreprod.110.087320 21084712 PMC3062034

[B28] Onofre J. Baert Y. Faes K. Goossens E. (2016). Cryopreservation of Testicular Tissue or Testicular Cell Suspensions: a Pivotal Step in Fertility Preservation. Hum. Reprod. Update 22, 744–761. 10.1093/humupd/dmw029 27566839 PMC5099994

[B29] Orth J. M. (1982). Proliferation of Sertoli Cells in Fetal and Postnatal Rats: a Quantitative Autoradiographic Study. Anat. Rec. 203, 485–492. 10.1002/ar.1092030408 7137603

[B30] Petersen C. Söder O. (2006). The Sertoli Cell - A Hormonal Target and 'Super' Nurse for Germ Cells that Determines Testicular Size. Horm. Res. Paediatr. 66, 153–161. 10.1159/000094142 16804315

[B31] Sharpe R. Mckinnell C. Kivlin C. Fisher J. (2003). Proliferation and Functional Maturation of Sertoli Cells, and Their Relevance to Disorders of Testis Function in Adulthood. Reproduction 125, 769–784. 10.1530/rep.0.1250769 12773099

[B32] Sharpe R. M. Walker M. Millar M. R. Atanassova N. Morris K. Mckinnell C. (2000). Effect of Neonatal Gonadotropin-Releasing Hormone Antagonist Administration on Sertoli Cell Number and Testicular Development in the Marmoset: Comparison with the Rat1. Biol. Reprod. 62, 1685–1693. 10.1095/biolreprod62.6.1685 10819772

[B33] Shetty G. Mitchell J. M. Wu Z. Phan T. T. (2020). Restoration of Functional Sperm Production in Irradiated Pubertal Rhesus Monkeys by Spermatogonial Stem Cell Transplantation. Andrologia 8, 1428–1441. 10.1111/andr.12807 PMC7521830 32351003

[B34] Singh D. Paduch D. A. Orwig K. E. Bolyakov A. (2017). The Production of Glial Cell Line-Derived Neurotrophic Factor by Human Sertoli Cells Is Substantially Reduced in Sertoli Cell-Only Testes. Hum. Reprod. 32, 1108–1117. 10.1093/humrep/dex061 28369535 PMC6075567

[B35] Smart E. Lopes F. Rice S. Nagy B. Anderson R. A. Mitchell R. T. (2018). Chemotherapy Drugs Cyclophosphamide, Cisplatin and Doxorubicin Induce Germ Cell Loss in an *In Vitro* Model of the Prepubertal Testis. Sci. Rep. 8, 1773. 10.1038/s41598-018-19761-9 29379115 PMC5788858

[B36] Tharmalingam M. D. Matilionyte G. Wallace W. H. B. Stukenborg J.-B. Jahnukainen K. Oliver E. (2020). Cisplatin and Carboplatin Result in Similar Gonadotoxicity in Immature Human Testis with Implications for Fertility Preservation in Childhood Cancer. BMC Med. 18, 374. 10.1186/s12916-020-01844-y 33272271 PMC7716476

[B37] Thomson A. B. Campbell A. J. Irvine D. S. Anderson R. A. Kelnar C. J. Wallace W. H. B. (2002). Semen Quality and Spermatozoal DNA Integrity in Survivors of Childhood Cancer: a Case-Control Study. The Lancet 360, 361–367. 10.1016/s0140-6736(02)09606-x 12241775

[B38] U.-C. Hipler, B. Hochheim, B. Knöll U. C. Hochheim B. Knoll B. Tittelbach J. Schreiber G. (2001). Serum Inhibin B as a Marker for Spermatogenesis. Arch. Androl. 46, 217–222. 10.1080/01485010151096540 11339648

[B39] Utriainen P. Suominen A. Mäkitie O. Jahnukainen K. (2019). Gonadal Failure Is Common in Long-Term Survivors of Childhood High-Risk Neuroblastoma Treated with High-Dose Chemotherapy and Autologous Stem Cell Rescue. Front. Endocrinol. 10, 555. 10.3389/fendo.2019.00555 PMC6694459 31440211

[B40] Vergouwen R. P. F. A. Jacobs S. G. P. M. Davids J. A. G. de Rooij D. G. De Rooij D. (1991). Proliferative Activity of Gonocytes, Sertoli Cells and Interstitial Cells during Testicular Development in Mice. Reproduction 93, 233–243. 10.1530/jrf.0.0930233 1920294

[B41] Yang Q. E. Kim D. Kaucher A. Oatley M. J. Oatley J. M. M. (2013). CXCL12-CXCR4 Signaling Is Required for the Maintenance of Mouse Spermatogonial Stem Cells. J. Cel Sci 126, 1009–1020. 10.1242/jcs.119826 PMC4074255 23239029

[B42] Zhang Z. Shao S. Meistrich M. L. L. (2007). The Radiation-Induced Block in Spermatogonial Differentiation Is Due to Damage to the Somatic Environment, Not the Germ Cells. J. Cel. Physiol. 211, 149–158. 10.1002/jcp.20910 17167785

