# Supplementary figures and images for: Maintenance of Sertoli Cell Number and Function in Immature Human Testicular Tissues Exposed to Platinum-Based Chemotherapy—Implications for Fertility Restoration
Source: Front Toxicol. 2022 Mar 21;4:825734. doi: 10.3389/ftox.2022.825734 (PMC8977418; doi:10.3389/ftox.2022.825734)

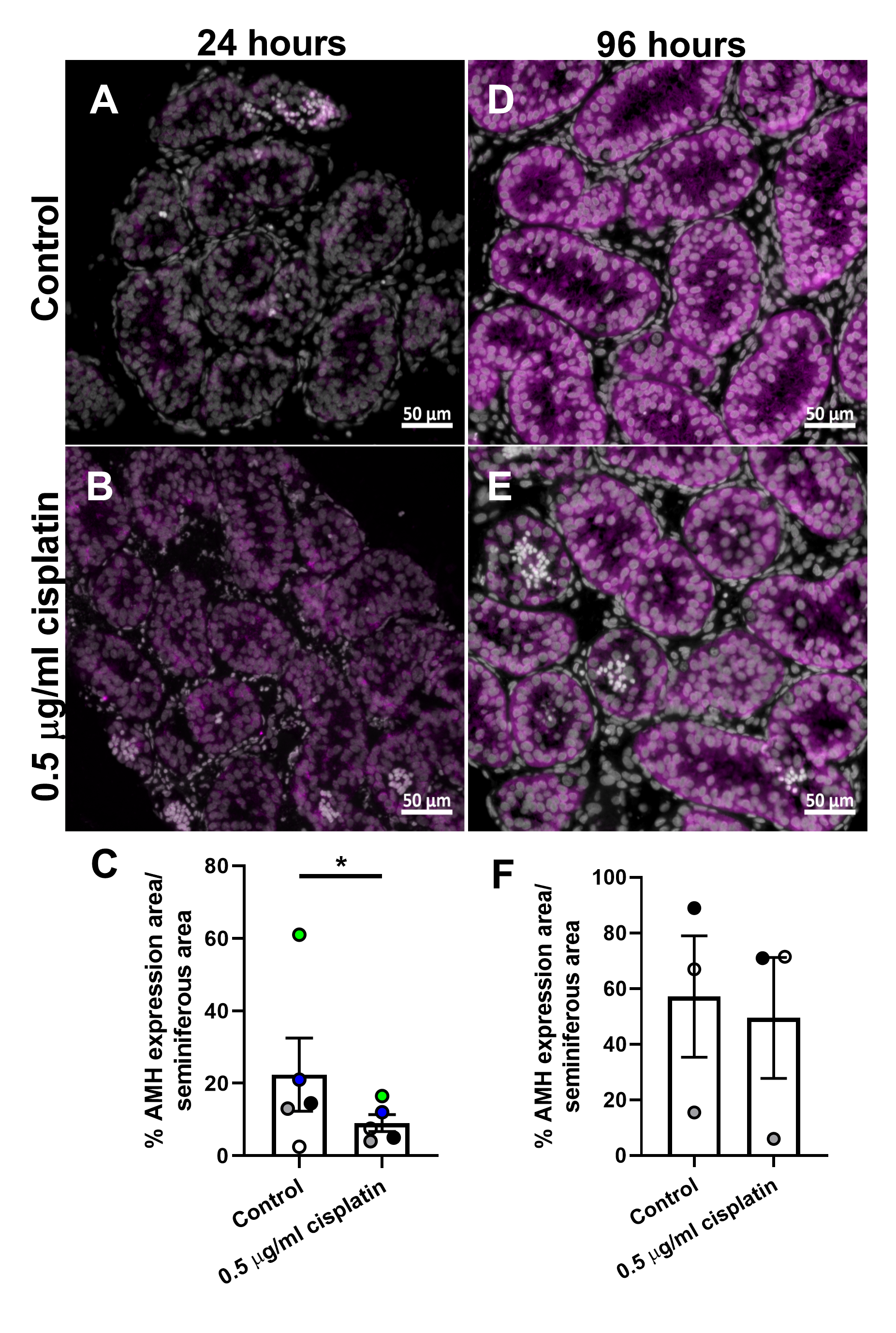

Supplement: Supplementary file 1 [file Image1.tif]
